# Removal of hexavalent chromium from wastewater by chelating resin supported Fe/Cu bimetallic nanoparticles: Characterization, performance and mechanisms

**DOI:** 10.1371/journal.pone.0318180

**Published:** 2025-03-18

**Authors:** Jialu Shi, Li Tang, Zhanhui Shen, Linan Deng, Xintong Liu

**Affiliations:** 1 Henan Key Laboratory for Synergistic Prevention of Water and Soil Environmental Pollution, School of Geographic Sciences, Xinyang Normal University, Xinyang, China; 2 Key Laboratory for Yellow River and Huai River Water Environment and Pollution Control, Ministry of Education, Henan Key Laboratory for Environmental Pollution Control, School of Environment, Henan Normal University, Xinxiang, China; 3 State Key Laboratory of Pollution Control and Resource Reuse, School of the Environment, Nanjing University, Nanjing, China; Konkuk University - Seoul Campus: Konkuk University, KOREA, REPUBLIC OF

## Abstract

In this work, Bimetallic Fe/Cu nanoparticles were successfully stabilized by chelating resin, which was specifically employed for the remediation of hexavalent chromium contaminated wastewater. Based on the characterization results, it was observed that the Fe/Cu bimetallic nanoparticles were uniformly and well distributed on the surface of the resin DOW M4195. The results demonstrated that the supported bimetallic Fe/Cu nanoparticles exhibited an excellent performance for Cr(VI) removal efficiency, reaching up to 99.4%. A series of factors, including initial pH, initial concentration of Cr(VI), co-exciting ions and humic acid were systematically evaluated to ascertain their respective impacts on Cr(VI) removal. The kinetics study followed intra-particle diffusion model demonstrated that both the adsorption and diffusion processes of Cr(VI) by the DOW M4195 resin played an important role in the overall removal of Cr(VI). The analytical results derived from XPS spectra at specific reaction times revealed the underlying removal mechanism of Cr(VI): Cr(VI) was adsorbed onto M-Fe/Cu due to the rich porous structure of the chelating resin DOW M4195. Additionally, the presence of the second metal, Cu, was found to significantly enhance the reduction performance of Fe^0^ and Fe(II) during the Cr(VI) removal process. The Cr(VI) removal mechanism was determined to involve a combination of physical adsorption, redox reactions and co-precipitation.

## 1. Introduction

Hexavalent chromium (Cr(VI)) is a typical toxic heavy metal ion that exists widely in industrial production processes such as processing of chromium ore, chemical manufacturing and electroplating [1]. The large amount of Cr(VI) containing wastewater and waste residue has led to serious Cr(VI) pollution in China’s natural water bodies. The methods to remove Cr(VI) include adsorption [2], ion exchange, membrane separation and so on. Although these methods are effective, there is a risk of releasing Cr(VI) into the environment.

Chromium predominantly exists in two valence states in the environment, Cr(VI) and Cr(III) [3]. Cr(VI) exhibits high mobile and pronounced toxicity in the environment, which is easily adsorbed by humans. Excessive exposure to substances containing Cr(VI) can substantially augment the cancer risk in humans. However, Cr(III) displays relatively weak migration and toxicity[4]. It is prone to hydrolysis in aqueous solution as the form of precipitates (Cr(OH)_3_ and Cr_2_O_3_) under alkaline or weakly acidic conditions [1]. Moreover, Cr(III) is also a necessary micro-precious element in the process of human metabolism [5]. Therefore, the conversion of highly toxic Cr (VI) into weakly toxic Cr (III) is one of the most effective approaches for controlling Cr (VI) pollution in water.

In recent years, nanoscale zero-valent iron (nZVI), which has high reducibility, environmental friendliness, strong absorption, low cost and easy to obtain[6], can achieve the reduction and fixation of Cr (VI) [7–9]. However, nZVI particles are susceptible to oxidize and agglomerate. The oxide layer of zero-valent iron can hinder the transport of electrons in the iron core, resulting in low activity of zero-valent iron. Many methods have been explored to enhance the activity of Fe^0^, including coupling Fe^0^ with magnetic field [10], combining Fe^0^ with strong oxidizer like H_2_O_2_ and persulfate [11, 12], as well as doping other metal elements onto the surface of Fe^0^. Among these methods, researchers have shown that doping an additional metal material on the surface of Fe^0^ has shown significant advantages, including simple preparation, low cost and high electron selectivity. Many kinds of metals are used for modifying nZVI, such as Fe/Ni [13], Fe/Cu [14], Fe/Pd [15], Fe/Au bimetallic and so on. Precious metals showed high efficiency for pollutant removal. However, the high cost of precious metals limits their application [16]. Copper, in particular, has been proven to promote the activity of zero-valent iron and is often used as an additional metal in combination with iron [17]. Compared with other bimetallic materials, Fe/Cu bimetallic nanoparticles has distinct properties. For instance, precious metal bimetallics such as Fe/Pd and Fe/Au, despite being effective in pollutant removal, are restricted by their high cost [13]. Fe/Cu bimetallic nanoparticles, on the other hand, present a more economical option without sacrificing reactivity. The potential between Fe^0^ and Cu^0^ (0.777 V), being higher than that between Fe^0^ and Fe^2+^ (0.441 V) [18], expedites the corrosion of Fe^0^ and thereby augments the reduction ability of the material.

Bimetallic nanoparticles are prone to agglomeration and oxidation during both the preparation and usage processes. Loading bimetallic nanoparticles on a carrier can effectively address the issue of agglomeration. Stabilizers such as carbon nanotubes (CNTs) [19], biochar [20], resin [21], carboxymethyl cellulose (CMC) [22] and grapheme [23] have been employed. In our prior study, the chelating resin was used as the carrier of metal catalyst, and the pyridine group on the resin could chelate with metal ions [24, 25]. The strong binding of the support to the M-NPs can enhance the stability of the M-NPs and ultimately improve their activity and service life. The porous characteristics of the resin material not only provides more sites for the loading of metal particles, but also provides favorable conditions for the diffusion of pollutants into the resin. The combination of Fe/Cu bimetallic nanoparticles and chelating resin has not been extensively studied, and this combination is expected to synergistically enhance the performance for Cr(VI) removal.

Many studies have investigated the mechanism of Cr(VI) removal [16,18,26]. Nevertheless, the proposed mechanism still requires verification. Further systematic research is needed to clarifying the actual role of Fe/Cu in the reduction of Cr(VI) and the reaction process of Cr(VI). It is crucial for the further advancements in Fe-based decontamination technologies. In this study, through XPS analysis during specific reaction times, a more detailed reaction mechanism will be revealed, especially focusing on the change of Fe/Cu chemical valence states, the role of Cu on contaminants and electron transfer, as well as the conversion of Cr(VI) to Cr(III) during the redox reaction, which have been neglected in previous studies. The investigation of the reaction kinetics of the chelating resin supported Fe/Cu bimetallic nanoparticles for Cr(VI) removal will provide valuable insights for the optimization and application of this material in the future.

The aim of this study was to (1) prepare chelating resin supported Fe/Cu bimetallic nanoparticles for Cr(VI) removal; (2) reveal the reaction mechanism according to the XPS analysis during specific reaction times; (3) investigate the reaction kinetics of the chelating resin supported Fe/Cu bimetallic nanoparticles for Cr(VI) removal.

## 2. Materials and methods

### 2.1 Materials and reagents

All chemicals used in this study are of analytical grade and were applied as delivered without further purification, including ferric sulfate (Fe_2_(SO_4_)_3_), copper chloride dehydrate (CuCl_2_·2H_2_O), potassium chromate (K_2_CrO_4_), ethyl alcohol, sodium borohydride (NaBH_4_), sodium nitrate (NaNO_3_), potassium dihydrogen phosphate (KH_2_PO_4_). The chelating resin DOW 3N (DOWEX™M4195) was purchased from Sigma Aldrich.

### 2.2 Synthesis of supported Fe^0^ and Fe/Cu bimetallic nanoparticles

The supported Fe^0^ nanoparticles and Fe/Cu bimetallic nanoparticles were prepared using a chemical reduction method with NaBH_4_ as the reducing agent. Briefly, 0.5 g DOW M4195 was added into the dissolved solution containing 50 mL 0.2 mol/L Fe^3 +^ solution and stirred for 30 min. Then, the resin spheres (DOW M4195-Fe^3+^) were washed several times with deionized water. Subsequently, 50 mL of a 4 wt.% NaBH_4_ solution was dropped slowly to reduce immobilized Fe^3 +^ to Fe^0^ under an N_2_ environment for 2 h at 20 ^0^C. The obtained black spheres M-Fe were rinsed multiple times with deionized water.

The supported Fe/Cu bimetallic nanoparticles were prepared using a similar method as described above for M-Fe synthesis but with some modifications: instead of only adding the dissolved solution containing 0.2 mol/L Fe^3 + ^, it also contained either 0.0066 mol/L, 0.01 mol/L or 0.02 mol/L Cu² ⁺ (the molar ratio between Fe/Cu being respectively set at: 3:1, 2:1, 1:1, respectively). The obtained black spheres were briefly named as M-Fe/Cu-1, M-Fe/Cu-2 and M-Fe/Cu-3, respectively.

### 2.3 Characterization of synthesized nanoparticles

The morphologies of Fe and Fe/Cu nanoparticles and the elemental composition were obtained using the scanning electron microscopy with energy dispersive X-ray spectroscopy (SEM-EDS, SU8010, Hitachi High-Technologies, Japan). The size and surface morphology of Fe and Fe/Cu nanoparticles were characterized using high-resolution transmission electron microscope (HR-TEM, JEM-2100) at 200KV with a resolution of 0.23 nm. The XPS spectras of the Fe and Fe/Cu before and after reaction were determined by an X-ray photoelectron spectrometer (XPS, ESCALAB 250Xi, Thermo Fisher, USA). The amount of Fe and Cu loaded into DOW M4195 and the released total iron ions and Cu^2 +^ ions concentrations were determined by inductively coupled plasma mass spectroscopy (ICP-MS, ELAN DRC-e, PerkinElmer, USA).

### 2.4 Batch experiments

Batch experiments of Cr(VI) removal by supported Fe and Fe/Cu nanoparticles were carried out in a conical flask with 200 mL 20 mg/L Cr(VI). A certain amount of composites with 0.5 g supports was added to investigate the activity of Fe and Fe/Cu nanoparticles. The conical flask were agitated at 25 ^0^C at 180 rpm. The initial of pH of Cr(VI) solution was adjusted using HCl or NaOH. The experiment was carried out under aerobic conditions. At specific time intervals, aqueous samples were taken out to determine the concentrations of Cr(VI) and total Cr after filtered through 0.45 μm millipore filters.

### 2.5 Analytical methods

Cr(VI) concentration was determined using 1, 5-diphenylcarbazide colorimetric spectrophotometric method by an UV-visible spectrophotometer at the wavelength of 540 nm. The total Cr concentration was analysed with atomic absorption spectrometry (AA700, PerkinElmer, USA).


$$\text{The concentration of Cr}\left(\text{III}\right)=\text{Ctotal Cr}–\text{CCr}\left(\text{VI}\right)$$ (1)

Where C_total Cr_ is the total Cr concentration and C_Cr(VI)_ is the Cr(VI) concentration.

## 3 Results and discussion

### 3.1 Reduction activity of M-Fe/Cu composites

**Fig 1:**
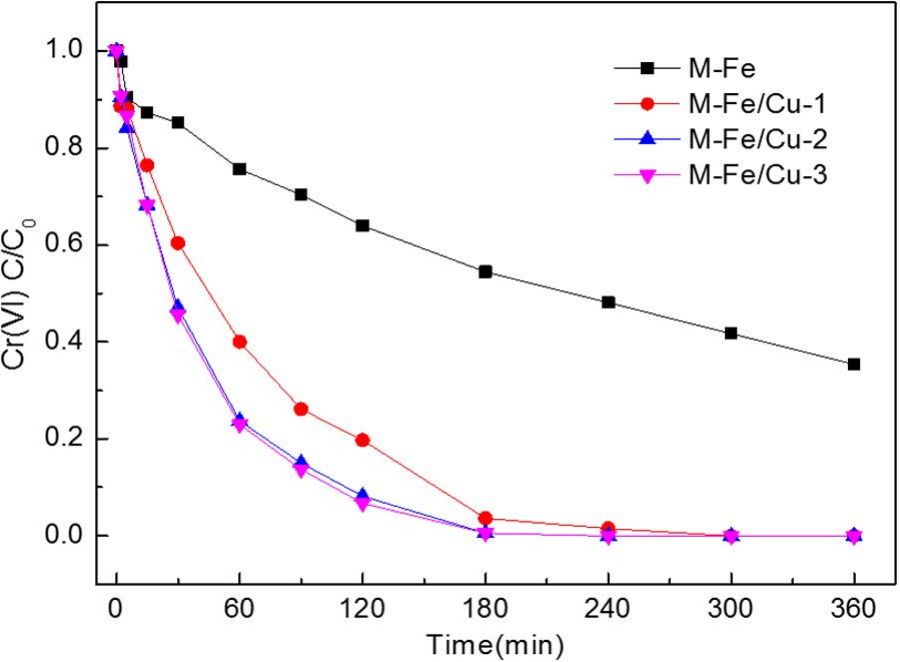
Cr(VI) removal performance by M-Fe and M-Fe/Cu with different Fe/Cu mass ratio (the initial concentration of Cr(VI): 20 mg/L, pH = 7, T = **25**^o^**C).**

The influence of Fe/Cu mass ratio on the removal efficiency of Cr(VI) was investigated and the results were presented in Fig 1. The performance of M-Fe in removing Cr(VI) was also investigated. The concentration of Cr(VI) was gradually declined as reaction time increased. The removal efficiency of Cr(VI) by M-Fe reached 64.6% at 6 h while the removal efficiency of Cr(VI) attained 96.4%, 99.4% and 99.4% at 3 h by M-Fe/Cu-1, M-Fe/Cu-2 and M-Fe/Cu-3, respectively. Significantly increased Cr(VI) removal rate was observed in M-Fe/Cu compared with M-Fe. The results showed that the incorporation of Cu could effectively improve the Cr(VI) removal performance of M-Fe. The reason of this phenomenon is generally considered that copper deposition can form galvanic battery and electron transfer layer [27, 28]. The removal efficiency of Cr(VI) improved slightly with the increasing of Cu coating (Fe/Cu molar ratio from 3:1 to 2:1). However, almost no difference in removal efficiency of Cr(VI) was observed when the Cu coating increased continuously (Fe/Cu molar ratio from 2:1 to 1:1). The best performance of M-Fe/Cu for Cr(VI) removal was M-Fe/Cu-2 and M-Fe/Cu-3. Considering lower Cu coating of M-Fe/Cu-2, the subsequent batch experiments were carried out with M-Fe/Cu-2 (Fe/Cu molar ratio was 2:1).

### 3.2 Characterization of Fe/Cu bimetallic nanoparticles

The loading amount of Fe and Cu on resin was shown in S1 Table. The Fe loading amount was 55.7 mg/g, and the Cu loading amount was 26.4 mg/g.

The surface morphology of M-Fe/Cu-2 was observed by SEM-EDS in S1 Fig. The resin DOW M4195 was spherical with a diameter of approximately 0.6 mm. The EDS analysis was found that Fe and Cu was homogeneously distributed on the surface of the resin DOW M4195 (S1 Fig (c) and (d)). Furthermore, a large amount of C (49.2%) and N (5.5%) was detected due to the resin DOW M4195 structure. The large amount of O (28.8%) element may be contributed by the oxidation of Fe and Cu during the process of preparation and vacuum drying. In comparison with DOW M4195 (S1 Fig (e)), there are some small particles on the surface of the resin DOW M4195 for M-Fe/Cu-2 due to Fe/Cu ladings (S1 Fig (f)). The abundant pore structure on the chelating resin DOW M4195 can be seen from S1 Fig (e) and (f).

The BET surface area of DOW M4195 and M-Fe/Cu-2 was shown in S2 Table. The results shown that after the loading of Fe and Cu on the chelating resin DOW M4195, the BET surface area was increased greatly. The pore-size distribution plot of DOW M4195 and M-Fe/Cu-2 before and after use was shown in S2 Fig. In comparison with DOW M4195, the pore volume in the range of 2-15 nm was obviously increased after loading Fe/Cu bimetallic nanoparticles, and thereby the BET surface area was increased. However, the pore-size distribution hardly changed before and after the reaction.

The microscopic morphologies of Fe/Cu nanoparticles were investigated by TEM and the images were displayed in S3 Fig. Black spherical Fe/Cu nanoparticles with a diameter of 5-10 nm were well-dispersed on the resin DOW M4195 which demonstrated that the resin DOW M4195 was a successful carrier helped to avoid the agglomeration of Fe/Cu nanoparticles.

S4 Fig presented the FTIR spectra of DOW M4195 and M-Fe/Cu-2 before and after reaction. In the FTIR spectrum of DOW M4195, four bands at 1589 cm^-1^, 1569 cm^-1^, 1433 cm^-1^ and 760 cm^-1^ were observed, which were associated with 2-picolylamine (the functional groups of the chelating resin DOW M4195). For the spectrum of M-Fe/Cu-2, the other new absorption peaks in the range of 2200–2400 cm^-1^ and at 3209 cm^-1^ could be ascribed to the bending vibration of B–O. This B–O was the residue left after the reduction of Fe/Cu with NaBH_4_. There was no significant change in the FTIR spectra of M-Fe/Cu-2 before and after the reaction of reducing Cr(VI).

**Fig 2:**
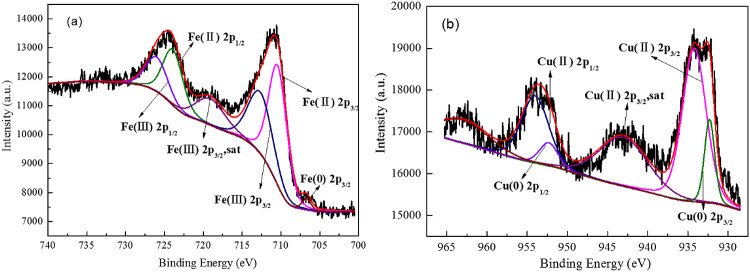
The XPS patterns of M-Fe/Cu-2: (a) Fe 2p and (b) Cu 2p.

XPS analysis were conducted to characterize the surface composition and chemical species of Fe/Cu bimetal nanoparticles and the results were shown in Fig 2. The peaks of Fe 2p at 710 eV and 725 eV corresponded to Fe(II) and the peaks of Fe 2p at 712 eV and 727 eV corresponded to Fe(III). Meanwhile, the peak at 707 eV is the characteristic peak of Fe (0). The results of XPS patterns of Fe 2p revealed that the iron oxide existed on the surface of Fe (0). As shown in Fig 2(b), The Cu 2p peaks at 954.6 eV, 943.2 eV and 934.8 eV represent Cu(II). The intense peaks of Cu 2p at 952.3 eV and 932.5 eV were corresponding to Cu (0), which demonstrated that Cu(0) was successfully impregnated in the resin DOW M4195.

### 3.3 Factors affecting on the Cr(VI) reduction

#### 3.3.1 Effect of initial pH on Cr(VI) reduction.

**Fig 3:**
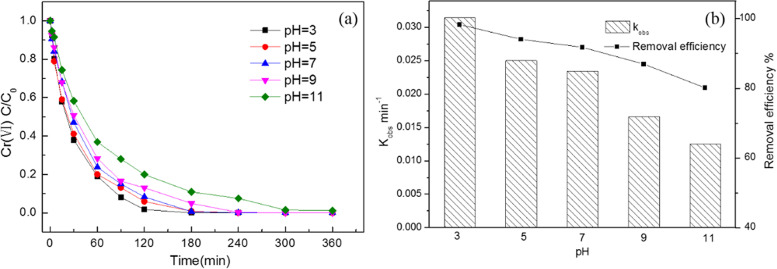
The effect of initial pH on the removal of Cr(VI) (a); the k_obs_ and the removal efficiency at 2 h (b) (the initial concentration was 20 mg/L Cr(VI), T = **25**^o^**C).**


The pH value play a critical role in Cr(VI) removal. The effect of initial pH on the removal of Cr(VI) by M-Fe/Cu-2 was presented in Fig 3. The kinetic model was used to describe the kinetics of Cr(VI) reduction. The results showed that Cr(VI) reduction kinetics accorded with the pseudo-first-order kinetic model. The pseudo-first-order kinetic model was shown below:

$$\frac{d\left[Cr\left(VI\right)\right]}{dt}=-K_{obs}\left[Cr\left(VI\right)\right]$$ (2)

$$\ln\left(\frac{\left[Cr{\left(VI\right)}_{0}\right]}{\left[Cr{\left(VI\right)}_{t}\right]}\right)=K_{obs}t$$ (3)

Where [Cr(VI)]_t_ is the concentration of Cr(VI) (mg L^-1^) at time t, [Cr(VI)]_0_ is the initial concentration of Cr(VI) (mg L^-1^), k_obs_ is the observed first-order rate constant (min^-1^).

The results showed that the initial pH had a significant impact on Cr(VI) removal. M-Fe/Cu-2 has a higher removal efficiency at lower pH. The removal efficiency of Cr(VI) declined slightly as the initial pH value increased from 3 to 9. However, with the increasing of initial pH from 9 to 11, the decrease amplitude of Cr(VI) removal efficiency was greater. The k_obs_ was also decreased as the initial pH increased. The results indicated that the reduction process of Cr(VI) was highly dependent on the initial pH, and the acidic conditions were favorable for the removal of Cr(VI).

S5 Fig showed the pHpzc diagram of M-Fe/Cu-2. It can be seen from the figure that the pHpzc of M-Fe/Cu-2 is approximately 7.8. When the pH of the solution is greater than 7.8, the surface potential of the material is negative, which is not conducive to the adsorption and diffusion of Cr(VI). Therefore, the removal efficiency and rate of Cr(VI) decrease under the conditions of pH 9 and 11. Conversely, when the pH of the solution is less than 7.8, the surface potential of the material is positive, which is conducive to the adsorption and diffusion of Cr(VI) and thus beneficial for the removal of hexavalent chromium.


$$3Fe^{0}+Cr_{2}O_{7}^{2-}+14H^{+}\to Cr^{3+}+3Fe^{2+}+7H_{2}O$$ (4)

$$6Fe^{2+}+Cr_{2}O_{7}^{2-}+14H^{+}\to 2Cr^{3+}+6Fe^{3+}+7H_{2}O$$ (5)

Eqs. (4)–(5) showed that all the reactions of hexavalent chromium reduction by Fe/Cu nanoparticles consumed H^+ ^. This explained the reason why acidic conditions were favorable for the removal of Cr(VI). In addition, high pH values may lead to a passivation layer on the surface of Fe/Cu nanoparticles, which inhibited the reduction of Cr(VI).

#### 3.3.2 Effect of initial concentration of Cr(VI) on Cr(VI) reduction.

**Fig 4:**
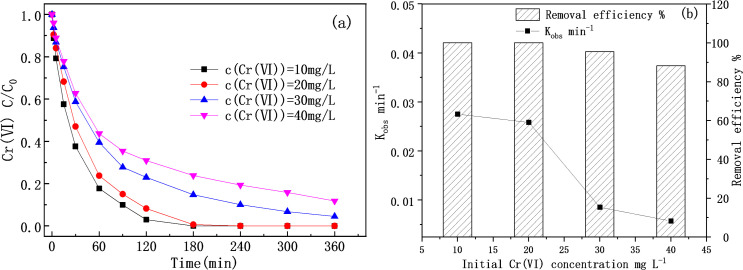
The effect of initial Cr(VI) concentration for the Cr(VI) reduction: the removal efficiency at 2 h (a) and the k_obs_ (b) (pH = **7, T** = **25**^o^**C).**

The effect of initial concentration on the removal of Cr(VI) by M-Fe/Cu-2 was shown in Fig 4. It was obvious that the removal efficiency of Cr(VI) declined slowly with the increase of the initial Cr(VI) concentration. Approximately 100% of Cr(VI) was eliminated within the first 3 h of the reaction when the initial Cr(VI) concentration was 10 or 20 mg/L. however, the removal efficiency was decreased when the initial Cr(VI) concentration increased to 30 or 40 mg/L. Similar to our results, Alessio Siciliano et al. also observed a slower Cr(VI) reduction with the increase of initial Cr(VI) concentration [29]. The decrease of removal efficiency may be ascribed to the coverage of excess Cr on the active sites of Fe/Cu nanoparticles. Moreover, the enhanced passivation of Fe/Cu nanoparticles at higher initial concentrations may also contribute to the lower efficiency. The reaction kinetics followed a first-order kinetic law with correlation coefficients R^2^ ranging between 0.928 and 0.989.

#### 3.3.3 Effect of co-exciting ions and HA on Cr(VI) reduction.

This study investigated the effect of co-exciting ions (NO_3_^-^, HCO_3_^-^, SO_4_^2-^, Mg^2 +^ and Ca^2+^) and humic acid (HA) on Cr(VI) reduction by M-Fe/Cu-2. The results were shown in S6 Fig and S3 Table. The results indicated that the co-existing anions such as NO_3_^-^, HCO_3_^-^, SO_4_^2-^ had obvious inhibitory effect on the reduction of Cr(VI). This phenomenon mainly because the co-existing anions can react with Fe/Cu bimetallic nanoparticles or be adsorbed onto the surface of Fe/Cu oxides. It may also be attributed to their ability to compete with Cr(VI) for the active site on the surface of M-Fe/Cu-2 composite [30]. Moreover, with the increase of the co-existing anions concentration, the Cr(VI) removal rate decreased obviously. In contrast, in the presence of co-existing cations Mg^2+^ and Ca^2+ ^, a different phenomenon was observed. The co-existing cations did not inhibit the removal of Cr(VI). Almost no change was observed in the removal of Cr(VI) in presence of Ca^2 + ^. Furthermore, Mg^2 +^ promoted the removal of Cr(VI) slightly. This is consistent with previous research results [31]. HA had obvious inhibitory effect on the removal of Cr(VI). The removal efficiency of Cr(VI) and K_obs_ decreased with the increasing concentration of HA. This was mainly because HA can be adsorbed onto the surface of the Fe/Cu nanoparticles and occupied the active sites [32].

### 3.4 Kinetic studies for Cr(VI) removal

Due to the porous structure of M4195 resin, the diffusion process of Cr(Ⅵ) may significantly impact the removal of Cr(VI). To understand the rate-determining step in this process, the intra-particle diffusion model was introduced.

The intra-particle diffusion model can be expressed by the following equation:


$$q_{t}=k_{p}t^{0.5}+C$$ (6)

Where, k_p_ is the diffusion rate constant (mg g^-1^ min^-0.5^); q_t_ is the adsorption amount of Cr(Ⅵ) by the composite material at different reaction time t (mg/g); C is the parameter of intra-particle diffusion model (mg/g).

The resulting fitted curve was shown in S7 Fig. The adsorption process of Cr(Ⅵ) by M-Fe/Cu-2 involved migration from the outer surface to the inner surface of the composite, which could be partitioned into three stages. The first stage is characterized by surface diffusion stage of Cr(Ⅵ). In this stage, Cr(VI) diffused from solution onto the surface of M-Fe/Cu-2. Due to a large number of active sites on the surface of the material, rapid Cr(Ⅵ) adsorption occurred with a high diffusion rate constant k_p1_. The second stage was the intra-particle internal diffusion, as adsorbed Cr(VI) gradually diffused from the surface of the material towards the interior pores of the material. Simultaneously, nZVI played an important role in reducing Cr(VI). The third stage corresponded to an adsorption equilibrium phase. At this point, adsorption reached equilibrium due to low concentration of Cr(VI) in solution and the accumulation of Fe(Ⅲ)-Cr(Ⅲ) precipitates generated by the reaction leading to decreased availability of active site on M-Fe/Cu-2. Consequently, both the adsorption and diffusion of Cr(VI) by the DOW M4195 resin played an important role in the removal of Cr(VI).

### 3.5 Reaction mechanism

To investigate the adsorption of DOW M4195 resin for Cr(VI), Cr(VI) removal by the resin M4195 was investigated. As depicted in S8 Fig, after 360 min, 23.1% removal efficiency was achieved without any detection of Cr(III). These results indicated that besides acting as support for Fe/Cu nanoparticles, M4195 resin also acted as an effective adsorbent for removing Cr(VI).

Furthermore, the activity of M-Fe/Cu-2 in the reduction of Cr(VI) was investigated in S9 Fig. It was observed that the concentration of Cr(Ⅵ) in the solution decreased continually, while the concentration of Cr(Ⅲ) increased gradually and reached its peak value at 60 min, indicating the conversion of Cr(VI) to Cr(Ⅲ). Simultaneously, the decrease in both Cr(Ⅲ) and total chromium concentration suggested that adsorption or co-precipitation of Cr(VI) and Cr(III) may occur on the surface of M-Fe/Cu-2.

**Fig 5:**
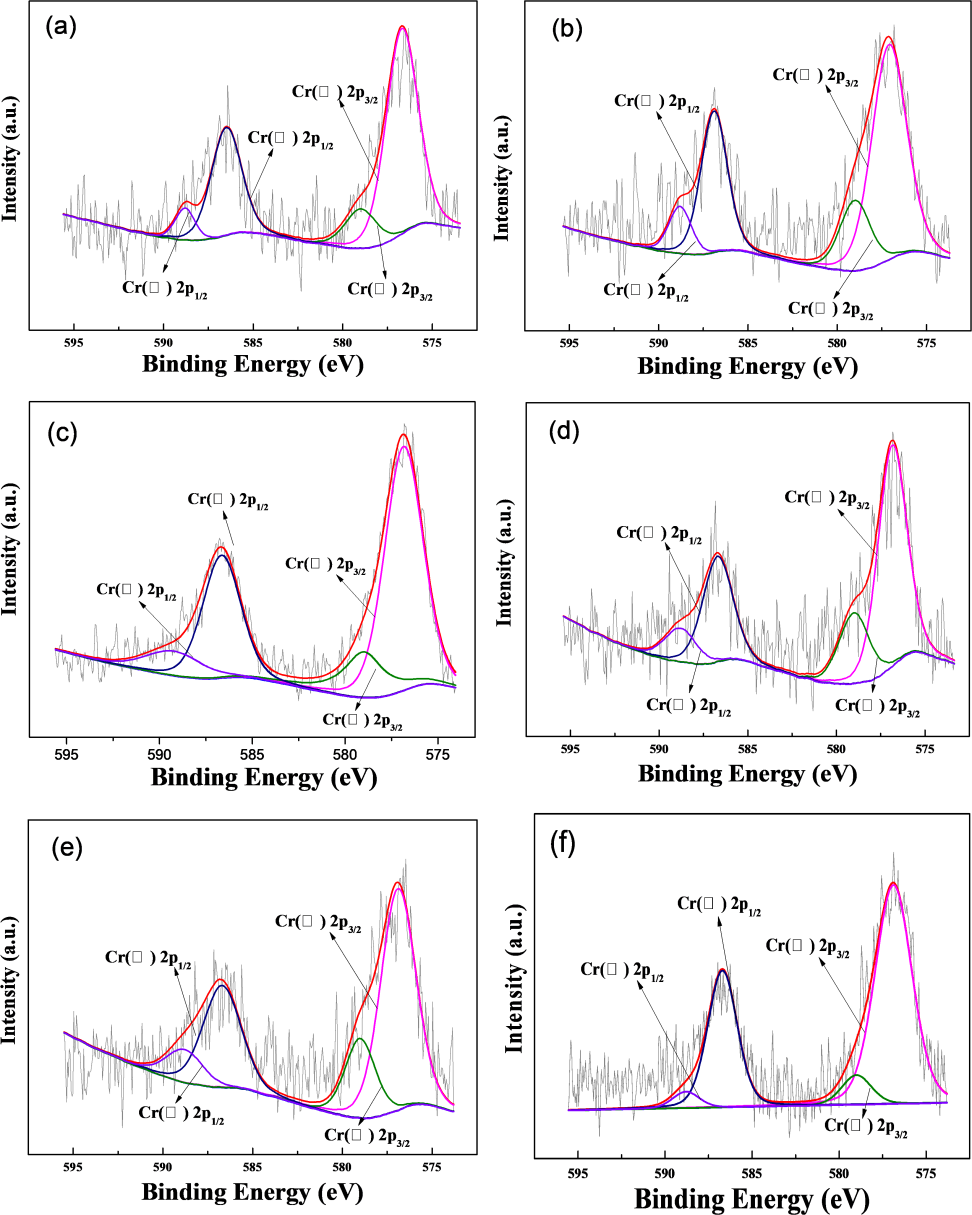
Cr 2p XPS analysis of M-Fe/Cu-2 after reaction for different time: (a) 5 min, (b) 15 min, (c) 60 min, (d) 90 min, (e) 180min, (f) 360 min.

In order to elucidate the reaction mechanism of Cr(VI), Cr 2p XPS analysis was conducted on used M-Fe/Cu-2 (Fig 5 (f)). The XPS spectra of Cr 2p region revealed coexistence of both Cr(VI) and Cr(III) on the surface of M-Fe/Cu-2 due to the strong peaks observed at 576.9 eV and 586.7 eV for Cr(III), as well as at 579.0 eV and 588.8 eV for Cr(VI). The results indicated that adsorption played a crucial role in capturing Cr(VI) by chelating resin DOW M4195 (as illustrated in S8 Fig), followed by subsequent reduction of a fraction of the adsorbed Cr(VI) to Cr(III) due to the presence of Fe-Cu bimetallic nanoparticles.

**Fig 6:**
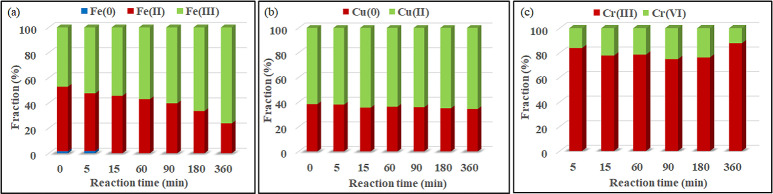
Fractions of Fe, Cu and Cr with different valence states in specific reaction time during the progress of Cr(VI) removal by M-Fe/Cu-2 according to the results of XPS spectra: (a) Fe, (b) Cu, (c) Cr.

The process of Cr(VI) reduction was further investigated through XPS analysis of M-Fe/Cu-2 at specific reaction time (Fig 5 and Fig 6 (c)). The results demonstrated an initial decrease followed by an increase in the proportion of Cr(III) during the redox reaction. Conversely, the proportion of Cr(VI) exhibited precisely the opposite trend. This phenomenon can be explained by the fact that, in the initial stage, Cr(VI) is primarily adsorbed on the surface of M-Fe/Cu-2 and quickly converted to Cr(III). During this stage, the adsorption rate of Cr(VI) is higher than the reduction rate, resulting in an elevation in Cr(VI) content and a diminution of Cr(III) content on the surface of M-Fe/Cu-2. With the progression of the reduction reaction, the concentration of Cr(VI) in the solution decreased rapidly, and the content of Cr(VI) adsorbed by M-Fe/Cu-2 also declined, simultaneously being converted to Cr(III). This finding was consistent with the results of intraparticle diffusion kinetic fitting. Therefore, in the later stages of the reaction, the content of Cr(III) gradually increased. The binding energies at 576.7 eV and 586.3 eV correspond to Cr(III) species such as Cr_2_O_3_, Cr(OH)O and Cr(OH)_3_ [33], indicating that the generated Cr(III) was co-precipitated on the surface of M-Fe/Cu-2. The adsorption and co-precipitation processes lead to a reduction in the total chromium concentration in the solution. Therefore, it can be inferred that the mechanism of Cr(VI) reduction may involve adsorption, reduction and co-precipitation.

**Fig 7:**
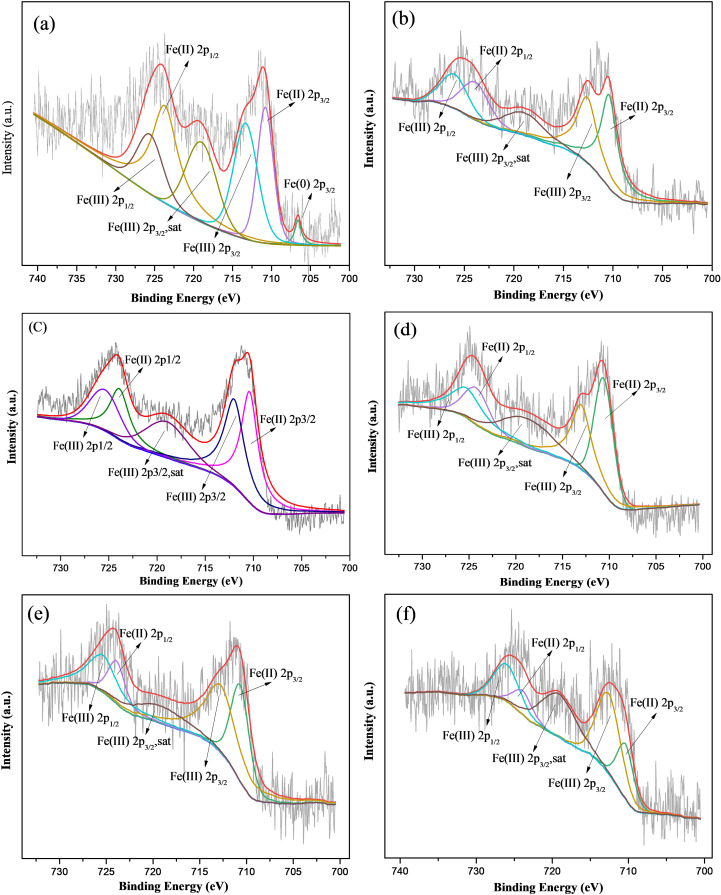
Fe 2p XPS analysis of M-Fe/Cu-2 after reaction for different time: (a) 5 min, (b) 15 min, (c) 60 min, (d) 90 min, (e) 180min, (f) 360 min.

Compared to fresh M-Fe/Cu-2, the XPS spectra of Fe 2p showed that the Fe^0^ peak disappeared in used M-Fe/Cu-2 after the removal process, demonstrating a transformation from Fe^0^ to Fe(II) or Fe(III) during the process of Cr(VI) reduction. Significant differences were observed when comparing changes in peak area proportion before and after reaction (as shown in Fig 7 and Fig 6 (a)). The proportion of Fe^0^ gradually decreased and completely disappeared within 15 minutes of the reaction. As the reaction advanced, the proportion of Fe(II) gradually declined while the proportion of Fe(III) increased. This indicated that both Fe^0^ and Fe(II) can be used to reduce Cr(VI). During the reaction, Fe^0^ was oxidized either to Fe(II) or/and Fe(III), and furthermore, Fe(II) was further oxidized to Fe(III).

**Fig 8:**
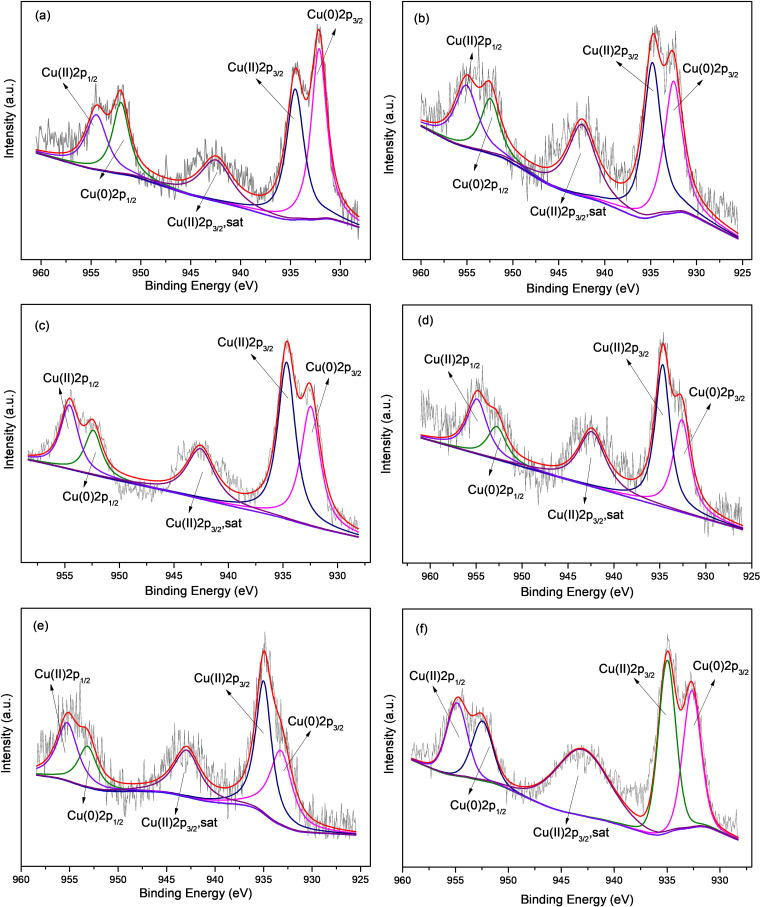
Cu 2p XPS analysis of M-Fe/Cu-2 after reaction for different time: (a) 5 min, (b) 15 min, (c) 60 min, (d) 90 min, (e) 180min, (f) 360 min.

The XPS spectra of Cu 2p showed no significant changes in position and intensity of Cu peaks before and after the reaction as shown in Fig 6 (b) and Fig 8. The results indicated that Cu did not directly reduce Cr(VI), but rather provided an active site for electron transfer, consistent with previous study [14].

**Fig 9:**
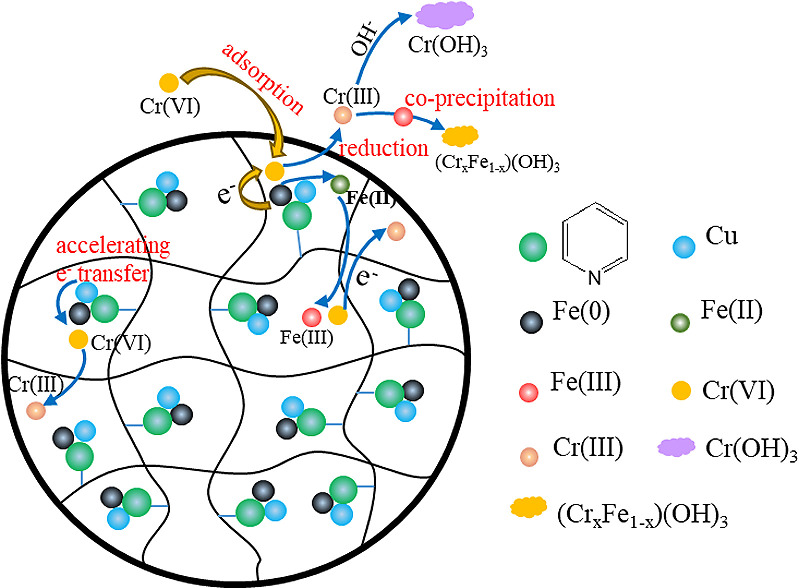
Schematic diagram of Cr(VI) removal by M-Fe/Cu-2.


In conclusion, XPS analysis revealed that the removal of Cr(VI) by M-Fe/Cu-2 material mainly involved physical adsorption, redox reactions and co-precipitation mechanisms. The mechanism of Cr(VI) removal by M-Fe/Cu-2 was shown in Fig 9. The mechanism can be summarized as follows: in the initial phase of the reaction, when the concentration of Cr(VI) in the solution was relatively high, there is obvious adsorption onto M-Fe/Cu-2 material. Nano zero-valent iron on the material, catalyzed by Cu, reduced Cr(VI) to form Cr(Ⅲ) and Fe(II). The contact between nano zero-valent iron and liquid phase leads to formation of Fe(II) and H_2_. Subsequent reactions between generated Fe(II) and Cr(VI) result in formation of Cr(III) and Fe(III). As time elapsed, the pH value continuously ascended, prompting a gradual transformation from Cr(III) and Fe(III) to Cr(OH)_3_, Fe(OH)_3_ or Fe(III)-Cr(III) co-precipitation. The equation for this reaction mechanism for M-Fe/Cu-2 material in removing Cr(VI) are presented as follows:

$$\text{3Fe}+{\text{2CrO}}_{\text{4}}{}^{\text{2}-}+{\text{16H}}^{\text{+}}\to 3{\text{Fe}}^{2+}+2{\text{Cr}}^{3+}+4{\text{H}}_{2}\text{O}$$ (7)

$$\text{Fe}+2{\text{H}}^{+}\to {\text{Fe}}^{2+}+{\text{H}}_{2}↑$$ (8)

$$3{\text{Fe}}^{2+}+{\text{CrO}}_{4}{}^{2-}+8{\text{H}}^{+}\to 3{\text{Fe}}^{3+}+{\text{Cr}}^{3+}+4{\text{H}}_{2}\text{O}$$ (9)

$${\text{Cr}}^{3+}+3{\text{OH}}^{-}\to \text{Cr}{\left(\text{OH}\right)}_{3}↓$$ (10)

$${\text{Fe}}^{3+}+3{\text{OH}}^{-}\to \text{Fe}{\left(\text{OH}\right)}_{3}↓$$ (11)

$${\text{xCr}}^{3+}+\left(1-\text{x}\right){\text{Fe}}^{3+}+3{\text{OH}}^{-}\to \left({\text{Cr}}_{\text{x}}{\text{Fe}}_{1-\text{x}}\right){\left(\text{OH}\right)}_{3}↓$$ (12)

## 4. Conclusions

In this study, the supported Fe/Cu nanoparticles were synthesized for the removal of Cr(VI). It was observed that Cu loading can significantly enhance the activity of nZVI in Cr(VI) removal. With the loading of Cu, the removal efficiency was increased from 64.6% to 99.4%. A number of factors, including initial pH, initial concentration of Cr(VI), co-exciting ions and humic acid influenced the performance of Fe/Cu bimetallic nanoparticles. A lower pH and lower initial concentration of Cr(VI) benefitted for Cr(VI) removal, while co-exciting ions and humic acid had adverse impacts. The kinetics study following intra-particle diffusion model demonstrated that the adsorption and diffusion of Cr(VI) by the DOW M4195 resin played an important role in the removal of Cr(VI). The XPS analysis of M-Fe/Cu-2 at specific reaction time was performed to elucidate the Cr(VI) removal mechanism. The results revealed that the Cr(VI) removal mechanism mainly involved physical adsorption, redox reactions and co-precipitation.

## Supporting information

S1 Fig SEM images and EDS spectrum of M-Fe/Cu-2. (DOCX)

S2 Fig The pore-size distribution plot of DOW M4195 and M-Fe/Cu-2 before and after use. (DOCX)

S3 Fig TEM images of M-Fe/Cu-2. (DOCX)

S4 Fig The FTIR spectra of DOW M4195 and M-Fe/Cu-2. (DOCX)

S5 Fig The pHpzc of M-Fe/Cu-2. (DOCX)

S6 Fig Effect of co-excited irons or HA on Cr(VI) reduction. (DOCX)

S7 Fig Internal diffusion model for Cr(VI) removal by M-Fe/Cu-2. (DOCX)

S8 Fig The adsorption of Cr(VI) by DOW M4195 resin (M_resin_ = 0.5 g, the initial concentration of Cr(VI) = 20 mg/L, pH = 7). (DOCX)

S9 Fig The removal of Cr(VI) by M-Fe/Cu-2. (DOCX)

S1 Table The loading amount of Fe and Cu on resin. (DOCX)

S2 Table The BET surface area of DOW M4195 and M-Fe/Cu-2. (DOCX)

S3 Table Effect of co-excited irons or HA on Cr(VI) reduction: Kobs and R2. (DOCX)
