# Supplementary material for: Removal of hexavalent chromium from wastewater by chelating resin supported Fe/Cu bimetallic nanoparticles: Characterization, performance and mechanisms
Source: PLoS One. 2025 Mar 18;20(3):e0318180. doi: 10.1371/journal.pone.0318180 (PMC11918381; doi:10.1371/journal.pone.0318180)
Supplement: S2 Fig — (DOCX) [file pone.0318180.s002.docx]

Removal of hexavalent chromium from wastewater by chelating resin supported Fe/Cu bimetallic nanoparticles: characterization, performance and mechanisms

Jialu Shi^1,2,3^, Li Tang^2^, Zhanhui Shen^1,2,3^, Linan Deng^1^, Xintong Liu^1^

^1^Henan Key Laboratory for Synergistic Prevention of Water and Soil Environmental Pollution, School of Geographic Sciences, Xinyang Normal University, Xinyang, China

^2^Key Laboratory for Yellow River and Huai River Water Environment and Pollution Control, Ministry of Education, Henan Key Laboratory for Environmental Pollution Control，School of Environment, Henan Normal University, Xinxiang, China.

^3^State Key Laboratory of Pollution Control and Resource Reuse, School of the Environment, Nanjing University, Nanjing, China


S2 Fig. The pore-size distribution plot of DOW M4195 and M-Fe/Cu-2 before and after use
