# Supplementary material for: Removal of hexavalent chromium from wastewater by chelating resin supported Fe/Cu bimetallic nanoparticles: Characterization, performance and mechanisms
Source: PLoS One. 2025 Mar 18;20(3):e0318180. doi: 10.1371/journal.pone.0318180 (PMC11918381; doi:10.1371/journal.pone.0318180)
Supplement: S3 Table — (DOCX) [file pone.0318180.s012.docx]

Removal of hexavalent chromium from wastewater by chelating resin supported Fe/Cu bimetallic nanoparticles: characterization, performance and mechanisms

Jialu Shi^1,2,3^, Li Tang^2^, Zhanhui Shen^1,2,3^, Linan Deng^1^, Xintong Liu^1^

^1^Henan Key Laboratory for Synergistic Prevention of Water and Soil Environmental Pollution, School of Geographic Sciences, Xinyang Normal University, Xinyang, China

^2^Key Laboratory for Yellow River and Huai River Water Environment and Pollution Control, Ministry of Education, Henan Key Laboratory for Environmental Pollution Control，School of Environment, Henan Normal University, Xinxiang, China.

^3^State Key Laboratory of Pollution Control and Resource Reuse, School of the Environment, Nanjing University, Nanjing, China

Table S3 Effect of co-excited irons or HA on Cr(VI) reduction: K_obs_ and R^2^

| Co-exciting ion or HA | Concentration mg L^-1^ | K_obs_ min^-1^ | R^2^ |
| --- | --- | --- | --- |
| Control | | 0.026 | 0.960 |
| NO_3_^-^-N | 10 | 0.014 | 0.963 |
|  | 20 | 0.010 | 0.940 |
| HCO_3_^-^ | 100 | 0.008 | 0.988 |
|  | 200 | 0.006 | 0.985 |
| SO_4_^2-^ | 100 | 0.014 | 0.984 |
|  | 200 | 0.012 | 0.993 |
| Mg^2+^ | 100 | 0.036 | 0.990 |
|  | 200 | 0.035 | 0.996 |
| Ca^2+^ | 100 | 0.218 | 0.993 |
|  | 200 | 0.020 | 0.983 |
| HA | 5 | 0.007 | 0.993 |
|  | 10 | 0.006 | 0.993 |
